# A Review of Current Research and Knowledge Gaps in the Epidemiology of Shiga Toxin-Producing *Escherichia coli* and *Salmonella* spp. in Trinidad and Tobago

**DOI:** 10.3390/vetsci5020042

**Published:** 2018-04-17

**Authors:** Anil K. Persad, Jeffrey LeJeune

**Affiliations:** 1 School of Veterinary Medicine, Faculty of Medical Sciences, The University of the West Indies, St. Augustine, Eric Williams Medical Sciences Complex, Mount Hope, Trinidad and Tobago; 2 Food Animal Health Research Program, The Ohio Agricultural Research and Development Center, The Ohio State University, 1680 Madison Ave, Wooster, OH 44691, USA; Lejeune.3@osu.edu

**Keywords:** Shiga toxin, *Escherichia coli*, food safety, Caribbean, veterinary medicine, one health

## Abstract

*Salmonella* and Shiga toxin-producing *Escherichia coli* are two of the main causes of foodborne disease globally, and while they have been implicated as possible causes of foodborne disease within the Caribbean region, the actual incidence is unknown. Trinidad and Tobago, one of the larger countries in the Caribbean, has an estimated annual foodborne disease burden of over 100,000 cases and, similar to other countries, the etiology of most of these cases is unknown. Both pathogens can reside as part of the normal gastrointestinal microflora of many wild and domestic animals, with animals acting as reservoirs, spillover hosts, or dead-end hosts. Carriage in animal species can be asymptomatic or, in the case of *Salmonella* in particular, there may be clinical manifestation in animals, which resemble the disease seen in humans. In this review, we will focus on the epidemiology of these two foodborne pathogens in Trinidad and Tobago and identify any knowledge gaps in the published literature. The filling of this critical knowledge void is essential for the development and implementation of appropriate mechanisms to reduce the dissemination and transmission of these pathogens, not only in Trinidad and Tobago, but also in the wider Caribbean.

## 1. Introduction

Globally, there are an estimated 600 million cases of foodborne disease resulting in over 500,000 deaths annually [1]. Foodborne disease, defined as diseases which occur as a result of ingestion of foodstuffs contaminated with microorganisms or chemicals, is one of the major causes of diarrheal diseases, and poses a major health and economic burden to many Latin American and Caribbean countries [2]. Trinidad and Tobago (T&T), located in the Southern Caribbean, is one such country where foodborne disease affects almost 10% of the human population (1.3 million) annually, with an economic burden conservatively estimated to be in excess of US$ 135 million dollars [3]. The actual incidence and etiology of foodborne disease in T&T and most Caribbean countries is, however, unknown, due to the absence of proper surveillance or diagnostic programs necessary for detecting the occurrence of foodborne disease and/or identifying the responsible pathogen [2].

Foodborne disease can be attributed to enteric bacterial, viral, or parasitic pathogens, and chemical contaminants or biotoxins [4]. Within Latin America and the Caribbean, bacterial pathogens, such as *E. coli*, *Salmonella*, *Shigella*, and *Vibrio Parahaemolyticus* were responsible for almost 70% of the recorded foodborne disease outbreaks (9180) during the period 1993–2010 [2]. Meat and dairy products were the origin of 30% of the outbreaks, 10.6% originated from water, and vegetable sources accounted for almost 9% [2]. Many of these bacterial pathogens (e.g., Shiga toxin-producing *Escherichia coli* (STEC), *Salmonella*, *Campylobacter*, and *Listeria*) can be part of the resident enteric flora of animals and shed in animal feces.

Humans are infected either via consumption of food and water contaminated with the feces of these animals; via direct contact with the animal or its environment; or via person-to-person transmission. The infectious dose is dependent on the pathogen, for example the infectious dose for *Shigella* spp. can be as little as 10 colony forming units, while the infectious dose for certain *E. coli* pathoypes can be as much as 100,000 colony forming units [5]. The onset of clinical signs is variable, and infections can be asymptomatic or manifest with a spectrum of clinical signs, including mild to bloody diarrhea, fever, vomiting, and systemic disease. Infections can be self-limiting or become systemic, resulting in chronic disease and/or potentially life threatening complications. The young, elderly, and immune-compromised are usually at greatest risk of infection and developing complications.

Historically, most bacterial foodborne disease has been mainly associated with the consumption of meat and animal products, however the incidence of foodborne disease associated with vegetable consumption, especially leafy greens, has been increasing [6]. Fecal contamination of vegetables is of particular concern, since many vegetables are eaten raw or with minimal cooking, and this, coupled with the fact that post-harvest intervention strategies for decontamination are ineffective, makes contamination prevention of paramount importance [6,7]. In an effort to decrease the incidence of foodborne illness, many producers have adopted programs such as the ‘farm to fork’ approach, which encourages the implementation of pre-harvest practices (Good Agriculture Practices (GAPs)) and post-harvest measures, such as Hazard Analysis Critical Control Plans (HACCP), which are aimed at minimizing the risk of pathogen-contaminated produce entering the food chain [8,9,10,11].

Although bacterial foodborne disease affects a large section of the Caribbean population annually, there is a dearth of published literature focusing on pre-harvest food safety in T&T and the wider Caribbean. In this review, we will focus on two main bacterial causes of bacterial foodborne disease and the research gaps which exist in understanding the epidemiology of STEC and *Salmonella* in T&T.

## 2. Shiga Toxin-Producing *Escherichia coli*

Shiga toxin-producing *Escherichia coli* (STEC) is a Gram negative bacterium which can be part of the normal gastrointestinal microflora of many animals [12], and is estimated to cause over two million cases of disease globally each year, yet there are no reported cases of human disease in T&T [13]. Interestingly, despite this apparent absence of human illness attributable to STEC, evidence of STEC in humans was reported by Roopnarine et al. (2007), who reported that approximately 1.4% of dairy farmers’ feces sampled were positive for *stx* genes [14]. The virulence properties of these isolates were not reported, however, and despite an exhaustive literature search, there were no other published reports of STEC surveillance in the human population.

STEC in T&T was first reported by Adesiyun (1993) in beef and pork samples collected from fresh meat markets [15]. The first report of STEC in livestock was made in 1994, when 14% of dairy cattle, 11% of pigs, and 23% of sheep samples were positive for STEC [16]. In that study, however, the serogroups present were not identified, nor were virulence profiles evaluated. Within the Caribbean, one study in Grenada reported that only 1/70 goats tested positive for *E. coli* O157 [17]. The relatively low carriage of STEC reported in small ruminants in these two Caribbean countries is of note, since reports from other geographic locations have reported STEC prevalence in small ruminants of up to 100% [12]. STEC, including serogroup O157, has also been detected in raw bovine milk samples, collected from milk churns and bulk milk tanks [18,19]. STEC is rarely associated with mastitis, and its presence in raw milk is more indicative of poor milking hygiene protocols [20]. This is particularly disconcerting, since it is estimated that up to 35% of dairy farmers in T&T consume raw milk [18]. Pet dogs, including dogs reared on bovine farms in T&T, have also been demonstrated to shed STEC [14,21]. Approximately 9% of fecal samples from farm dogs residing on bovine farms tested positive for *stx* genes, however whether this was transient shedding or as a result of dogs being colonized was not determined. Neither the serogroups recovered from dogs nor the genetic relatedness of the isolates to those recovered from cows were reported [14]. *E. coli* O157 has also been isolated from oysters sold by roadside vendors [22]. Mammalian wildlife species, rats, and diarrheic foals have also been screened for STEC in subsequent studies, but none tested positive [23,24,25].

There is a dearth of published literature describing the role of non-animal sources in the transmission and propagation of STEC. An extensive literature search revealed only two published papers evaluating the microbial quality of drinking water, one evaluating vegetable contamination, and no reports on the microbial quality of irrigation water. One of the papers on the microbial quality of drinking water focused on urban drinking water and the other on rural drinking water. Quite disconcerting is that 67% of rural and 33% of urban water samples were contaminated with *E. coli* [26,27]. Even more startling is that 15% of the *E. coli* colonies recovered from the rural drinking water samples were *stx* positive and 2% were identified as being O157 colonies. None of the *E. coli* isolates recovered from the urban drinking water samples were reported as being *stx* positive or of the O157 serogroup. As for microbial contamination of vegetables, only one published report was found, and this evaluated ready-to-eat vegetable samples collected at supermarkets. Of the 71 samples screened, one sample was positive for *E. coli*, but the *stx* profile and virulence profile were not investigated [28].

## 3. *Salmonella enterica* Subspecies *Enterica*

*Salmonella enterica* are Gram negative, facultative anaerobic bacteria which can be harbored as part of the enteric flora of many animals and shed in the feces of these animals. The duration of animal *Salmonella* shedding can be variable, for example the median shedding time for cattle is reported to be 50 days, with a maximum shedding period of up to 391 days [29]. Once in the environment, *Salmonella* serotypes have also been demonstrated to persist for several months, even extending to over one year in some studies [30,31,32]. The survival of *Salmonella* in the environment can differ between serovars, and can be dependent on many factors, including the chemical characteristics, pH, and moisture content of soil and fecal material. *Salmonella* environmental persistence can also be dependent on interactions with other microorganisms in the environment [33].

Serovars of *Salmonella enterica* have been associated with foodborne disease globally for over 100 years [34]. The global incidence of *Salmonella* is estimated to be almost 100 million cases [35]. Within T&T, the annual incidence is estimated to be over five cases per 100,000 persons, and 1.7% of childhood diarrhea cases screened between 1998–2000 were *Salmonella* positive [36,37]. The largest outbreak of non-typhoidal human salmonellosis in T&T occurred in 1973, when over 3000 persons became ill as a result of the consumption of contaminated powdered milk [38]. Other sporadic outbreaks have since occurred, and were potentially linked to the consumption of contaminated food and water [39,40]. Most human *Salmonella* cases are self-limiting, however young children under five years, the elderly, and the immunocompromised are most at risk of becoming infected and developing complications [41,42].

The majority of published *Salmonella* research in T&T has focused on the role of poultry and table eggs as sources of infection of the human population [43,44,45,46]. Indar et al. (1998) reported that 1.2% of the internal contents and 4.7% of egg shells were contaminated with *Salmonella* [43]. Another survey in 2005, testing eggs sold in markets, revealed that 2.9% of egg shells and 8% of egg contents were positive for *Salmonella*. This same study reported that 18% of egg shells and 3.6% of egg contents were positive for *E. coli* [44]. Another, more recent, study reported that *Salmonella* was recovered from 60% of layer farms, and 12.5% of egg shells sampled. The egg contents were, however, all negative for *Salmonella* [47].

Historically, *Salmonella* Enteritidis has been the serovar most associated with poultry and egg contamination, but recent research has postulated the emergence of new serovars, including *Salmonella* Anatum and *Salmonella* Kentucky [47]. The risk of *Salmonella* transmission to humans from poultry in T&T is quite profound, as one study evaluating the prevalence of *Salmonella* in chicken carcasses sold by retail vendors reported that 80% of carcasses were positive for *Salmonella* [46,48]. This high retail *Salmonella* prevalence could be a result of the combination of two factors: (i) A high on-farm *Salmonella* prevalence in live broiler birds and (ii) poor slaughter and processing practices that allow for cross-contamination during processing [46,49]. The prevalence in live Muscovy ducks reared for human consumption was reported to be as high as 40%, based on PCR screening [50].

While poultry and eggs have been identified as potentially major sources of *Salmonella*, other livestock, pets, and wild animal species can also serve as reservoirs of *Salmonella*. Cazabon et al. (1978) first reported pork as a possible vehicle for *Salmonella* transmission in T&T, and in that study 18% of swine carcasses sampled at the abattoir were positive for *Salmonella* [51]. A more extensive abattoir study conducted in 1993 detected *Salmonella* in 3% of goat meat samples, but all pork, beef, mutton, and chicken samples screened were negative [15]. Despite goat meat being a delicacy in T&T, and having been identified as a vehicle for the transmission of *Salmonella*, there is only one published study investigating *Salmonella* epidemiology in goats. In that study, a very small sample size of ten goats was tested, and all tested negative for *Salmonella* [52]. Another cross-sectional study, conducted by Adesiyun et al. (1994), reported the *Salmonella* prevalence in cattle, pig, and sheep feces to be 5%, 4%, 3% respectively [16]. Among pets, *Salmonella* has also been detected in dogs (3.6%), cats (2.1%), pet birds (0.9%), and aquarium fish (0.4%) [53,54,55]. While various animal species have been identified as reservoirs of *Salmonella*, there are no published studies in Trinidad and Tobago describing the recovery of molecularly identical *Salmonella* isolates from either human or animal species, or environmental sources, thus the exact source of human *Salmonella* infections remains undetermined.

Similar to STEC, the role of vegetable produce in *Salmonella* epidemiology is a neglected sphere of *Salmonella* research. Vegetable produce contamination can occur in the same manner as STEC contamination [56], however there are no published literature in T&T investigating non-animal sources of *Salmonella*. There are also no published reports investigating *Salmonella* contamination of surface or ground water used for irrigation or drinking. Given the previous detection of *E. coli* in both urban and rural drinking water, it is plausible that these water sources may also be contaminated with *Salmonella*, [26,27]. Adding credence to water sources being contaminated with *Salmonella* is the fact that *Salmonella* has been recovered from ponds where commercial fish are reared. *Salmonella* has also been recovered from free-living aquatic shellfish, such as oysters harvested from mangrove swamps [22,57]. These swamps are usually the terminal point of many rivers and streams from which irrigation water is sometimes collected, raising the possibility that irrigation water may also be contaminated with *Salmonella*.

## 4. Knowledge Gaps in STEC and *Salmonella* Epidemiology

From the literature cited above, it is indisputable there are many knowledge gaps in the epidemiology of STEC and *Salmonella* in T&T which must be filled before one can fully comprehend the impact of these pathogens.

Firstly, the role of vegetable produce in the epidemiology of both of these pathogens remains a largely unexplored sphere in T&T. Vegetable contamination on the farm can occur via a number of routes, including wind dispersal of pathogens from nearby livestock farms, animals defecating in the production field, the use of improperly treated manure or biosolids, poor worker hygiene, the use of contaminated irrigation water, flooding, and contaminated equipment [56]. The potential for indirect contamination of vegetable fields and produce from livestock farms is without question. In a 1998 study conducted by the Environmental Management Authority of Trinidad and Tobago on water quality, all major ground and surface water sources were reportedly contaminated, or at risk of contamination, by effluents from housing and livestock farms [58]. Additionally, many vegetation plots in T&T are at risk of flooding on an annual basis, which can lead to further contamination of produce and produce fields from these contaminated surface water sources [59].

While the role of vegetables in foodborne disease is largely unexplored in T&T, the importance of vegetables in the epidemiology of foodborne disease pathogens is demonstrated in the United States, where over 50% of foodborne disease cases from 1998–2008 were attributed to plant commodities [60]. During this period, leafy vegetables alone were estimated to be responsible for almost 2.2 million disease cases, or 22% of all foodborne disease cases, the most of any food commodity [60]. Similarly in China, plant commodities were associated with 48% (930) of foodborne disease outbreaks which occurred from 2003–2008 [61]. There are currently no published reports identifying the food vehicles for the transmission of foodborne disease in T&T. However, a survey of dietary consumption patterns in T&T revealed that 38% of participants regularly ate mixed raw vegetable salad, and a further 22% reported eating raw lettuce or tomatoes, thus the risk of consumption of contaminated produce is very apparent [62]. This high consumption rate, coupled with low compliance of farmers to Good Agriculture Practices, makes vegetables an important risk factor for foodborne disease [63].

Another critical void is the absence of recent research focusing on livestock species used for meat consumption. There are an estimated 22,000 pigs, 20,000 cattle, 11,000 goats, 9000 sheep, and 1500 water buffalo reared in T&T and, with the exception of cattle and water buffalo, the rest are reared primarily for meat production [64,65,66,67]. Other than poultry, there is a dearth of recent research focusing on these livestock species. In fact, most available published research is at least fifteen years old. Since then there have been many changes to animal genetics, nutrition, management, and production systems, and the emergence of new diseases. One would expect that with these changes there would be concurrent changes to exposure potential and susceptibility to certain pathogens.

While cattle and buffalo are slaughtered at properly constructed abattoir facilities, other smaller species are mainly slaughtered using backyard/road-side facilities, in crudely constructed slaughter facilities, which are usually devoid of running water and proper sanitation protocols. Despite this appreciable risk, there is a paucity of STEC and *Salmonella* research focused on these animals, especially sheep and goats.

Another important observation from the review of the existing published literature is that diagnostic methods for STEC did not include the critical step of sample enrichment. Sample enrichment increases the sensitivity of diagnostics tests and is necessary, since STEC, when present in feces, is usually present in such lows numbers that it can be almost impossible to isolate via direct plating [68,69,70]. There is also the need to place increased focus on the detection of non-O157 STEC serotypes. With increasing diagnostic capabilities, non-O157 STEC pathotypes are now being identified as the cause of an increasing number of foodborne diseases [71]. Another critical epidemiological factor missing in existing STEC literature in Trinidad and the Caribbean is the absence of virulence profiling. Although some studies report the *stx* profile, most do not evaluate other virulence factors, such Intimin or Enterohemolysin [17,72,73,74].

From the review of the literature above, one can conclude that the lack of molecular typing of isolates is a critical gap that needs to be filled in order to fully comprehend the ecology and epidemiology of these two foodborne pathogens in T&T. The use of Whole Genome Sequencing (WGS), which is increasingly becoming more available and cheaper, is one method which can be employed to fill this void [75]. The superior molecular resolution offered by WGS compared to conventional PFGE will allow for more accurate identification and distinguishing of bacterial strains [76]. This can potentially allow the identification of clonal subtypes which may be more abundant in certain niches, or demonstrate potential host species specificity by bacterial strains [77,78].

## 5. Conclusions

Even with limited detection and surveillance programs, the incidence of foodborne disease in T&T continues to increase annually [79]. The reduction in foodborne disease can only be achieved with multifaceted projects which incorporate research, application, and education programs. From the literature cited, it is indisputable that there are many critical knowledge gaps in the epidemiology of foodborne pathogens in T&T. The goal of these research endeavors must not only be aimed at filling these gaps, but also include identifying and evaluating appropriate pre-harvest intervention mechanisms to reduce the transmission of foodborne pathogens. There is also a need for education programs which inculcate the importance for good agriculture practices, as well as safe post-harvest handling and preparation of food.
